# Capacity Planning of Virtual Wards for Frail and Elderly Patients

**DOI:** 10.3390/healthcare12050533

**Published:** 2024-02-23

**Authors:** Harriet Grange, Gemma Johns, Alka Ahuja, Paul Harper, Elizabeth Williams, Daniel Gartner

**Affiliations:** 1 School of Mathematics, Cardiff University, Cardiff CF24 4AG, UK; 2 National Health Service (NHS), Aneurin Bevan University Health Board, Caerleon NP18 3XQ, UK

**Keywords:** virtual wards, telemedicine, remote monitoring, operational research, demand management, supply management

## Abstract

This paper investigates the planning of virtual ward (VW) capacity including the remote monitoring of frail and elderly patients. The main objective is to optimize VW hub locations across a region in the United Kingdom. Furthermore, assigning the optimal number of clinicians to different regions needs to be considered. We develop a mathematical model that minimizes the setup and travel costs of VW hubs and staff. Our experimental analysis evaluates different levels of demand considering postcode areas within different Trusts, also known as Health Boards, in the National Health Service (NHS). Furthermore, our experiments provide insights into how many hub locations should be deployed and staffed. This can be used to individually find the number of remote monitors and clinicians for each facility as well as the system overall.

## 1. Introduction

The healthcare industry is constantly developing as a result of technological breakthroughs, evolving patient requirements, and the pursuit of effective and patient-centred services. In Wales, TEC Cymru, a national digital transformation programme supported by the Welsh Government and accommodated within the Aneurin Bevan University Health Board (ABUHB), was created in response to this technological progress in healthcare. Their main objective is to enable patients to access healthcare more flexibly and conveniently. TEC Cymru aims to expand a Virtual Ward system that has already been rolled out in Swansea Bay. To expand the VW across Wales would be a major operational, logistical, and strategic problem in terms of providing care for frail and elderly patients. With the use of different remote monitoring techniques including wearable technologies, applications, technological platforms, and medical equipment, VWs enable patients to receive professional treatment from the comfort of their homes or other places of residence [1]. These monitoring tools, along with the participation of multidisciplinary healthcare teams situated in the neighbourhood, provide ongoing assistance and individualised treatment for patients at a hospital level.

The main goal of this research is to create a mathematical model that allows health authorities in Wales to determine the location and capacity needed for VWs. The model includes geographical characteristics of patients across Wales and where best these VWs should be located to provide care; we also identify potential locations with access to services.

This study intends to assist strategic decision-making processes at both organisational and policy level by comprehending the patterns of VW uptake and identifying hurdles and enablers of adoption. The knowledge gathered will aid in optimising the distribution of healthcare funds, ensuring that VW services adequately meet the various requirements of various populations. The study could also significantly improve the quality and accessibility of treatment, therefore aiding patient satisfaction and improving the well-being of the population. This research intends to produce useful insights that will affect future healthcare planning and digital transformation projects through quantitative modelling and decision making.

### 1.1. Background

With the world population ageing at an accelerating rate, planning healthcare services for elderly and frail patients becomes increasingly important. With the elderly population increasing, it has been estimated that by 2050 over 21% of the global population will be aged 60 and over [2,3]. The older population is increasing; meanwhile, there is a reverse effect occurring in the working-age population, the number of whom is decreasing. There will only be 2 people of the working age for every 1 person over 65 in Europe by 2050 [4]. DFor this reason, there is uncertainty around the supply of continuous healthcare professionals, in particular qualified and skilled professionals able to meet the needs of elderly patients [5]. As a result, the demand for long-term healthcare, particularly for elderly and frail patients, is predicted to rise shortly. Subsequently, if digital home care proves to be significantly more cost-effective, the demand for this speciality of care will increase, relieving the strain on healthcare professionals in hospital care [6]. Thus, creating good foundations before it is released is essential, as thus far, the home healthcare industry has been confronted with a plethora of challenges related to capacity planning, cost-efficiency optimisation, and service quality, necessitating prudent decision-making processes [5].

### 1.2. Elderly and Frail Patients

Elderly patients are considered vulnerable as they have an increased risk of accidents and contracting infectious diseases. In 2021, it was predicted that between a half and a quarter of people over 85 were classified as frail [7]. Elderly and frail patients when admitted to hospital are at high risk of deconditioning. It was shown that patients over 80 years old are at risk of their muscles ageing 10 years after just 10 days in acute or community hospitals [8]. Additionally, it was found that 48% of patients over 85 years old pass away within a year of being admitted to hospital. Thus, treating and monitoring patients from the comfort of home could be beneficial by preventing them from being placed in a high-risk environment. Patients who cannot or do not want to use traditional medical facilities might benefit greatly from home health care and telemedicine. However, due to their social isolation, the older population and home health care patients may exhibit bad behaviours and suffer from detrimental health impacts [5]. The fact that patients may keep living at home in a comfortable environment greatly enhances their quality of life. The health care system can also save a substantial sum of money when hospitalisation costs are avoided.

Improving the allocation of resources in healthcare is an increasing concern due to the overall decrease in the public budget [9]. It has been proven that healthcare at home decreases the load on hospitals and improves access in rural areas [10]. Healthcare professionals have worked simultaneously together to obtain the best outcome when managing and providing support for the elderly, which has subsequently reduced the load on hospitals [8]. The challenge is to not increase the budget in comparison to traditional hospital care while attempting to access and deliver care to rural areas.

In numerous developed countries, healthcare facility costs have witnessed an upward trajectory due to the requirement of providing long-term and continuous healthcare support for the ageing population, as well as the need for palliative care to address the burden of chronic disease [5]. Although the healthcare trajectory of these patients follows a similar path to that of other individuals, a clear association exists between the level of frailty and the extended duration of recovery as well as more specialised care [11].

When compared to hospital care, home healthcare offers an economical alternative, with many European countries allocating a portion of their total health budget, typically ranging from 1% to 5%, to support healthcare services delivered at home [4]. Home healthcare notably demonstrates its effectiveness in reducing hospital readmissions by carefully scheduling the release of elderly hospital patients [12]. Additionally, the utilisation of hospital-at-home programmes results in decreased infection rates and the mitigation of adverse effects associated with hospital stays [13].

A variety of professionals are involved with home healthcare services. Patients are often assessed and assisted by nurses, but depending on the situation, they can also need additional professionals such as a physiotherapist, doctor, or psychologist. These patients or professionals might need to acquire and handle certain facilities at home in some circumstances. As a result, supplying home healthcare services requires a variety of resources, such as nurses and other types of operators, support personnel, and material resources [14]. Given how diverse home care is, decision making is exceedingly complicated. As described by Jørgensen et al. [4], it involves long-term care, short-term recovery following hospital release, and palliative care. In the study conducted by Lanzarone et al. [15], a stochastic model was employed to analyse the care pathway of patients receiving home healthcare. This model facilitated the prediction and estimation of patients’ requirements, enabling a proactive approach to resource planning and allocation within the home healthcare setting. In order to include the most vulnerable patients within the VW, the identification of high-risk individuals is aided through association with GP practices involved in the Swansea Bay pilot [16]. Within the Swansea Bay VW, they conduct weekly multidisciplinary meetings which include all levels of caregivers such as primary care, secondary care, and voluntary care. Nevertheless, a notable challenge arises concerning the management of an adequate and qualified healthcare workforce, as the recruitment and dismissal of personnel cannot be readily adjusted to accommodate mid-term variations in demand. Consequently, staff dimensioning emerges as a crucial decision-making process for healthcare providers, requiring careful consideration and planning to ensure optimal resource allocation and effective service delivery [10,17].

Not only is this care more beneficial for patients’ well-being, but research has shown that given a choice between care at home or in a hospital, the majority would favour home care and being treated in their own environment [18]. Within the Welsh population, 8% of those 60 and older have reported encountering prejudice that they believed to be potentially connected to their age, with an additional 8% of the older population being made to feel they are too old for healthcare services [19]. In Wales, around one in five elderly individuals are considered to live in relative poverty, which contributes to the challenges faced when utilising healthcare services. The VW model offers a way to meet these preferences and provide high-quality care in a home setting.

Remote patient monitoring programmes might be crucial in improving the quality of healthcare and alleviating strain on hospitals within the NHS. Technologies for remote patient monitoring are a type of digital health platform that allows for patient evaluation outside of a conventional clinical visit in the patient’s home or community [20]. Symptom questionnaires, wearable sensors, and other medical equipment are used in remote monitoring programmes to gather medical data, which are then sent to a healthcare professional for clinical assessment and analysis. In the current research, it was discovered that remote patient monitoring decreased mortality and re-admissions in patients with chronic obstructive pulmonary disease and heart failure [21]. Numerous studies conducted over the past ten years have shown how remote patient monitoring can help people with chronic health issues to perform better.

The complex nature of telemedicine in VWs requires efficient matching of supply and demand for healthcare services [22]. This has become an increasing concern in recent years as the demand for health and social care systems in most European nations is rising due to socioeconomic and demographic changes [23]. Studies have proposed intermediary intervention and robust assignment models to optimise the allocation of healthcare professionals and services in these contexts. These models consider uncertainties in service duration, patient no-show behaviours, and geographical constraints. By incorporating these factors, healthcare providers can enhance the efficiency and effectiveness of resource allocation, ultimately leading to improved patient outcomes and satisfaction. The integration of VWs into healthcare systems has shown promising results. Several case studies have highlighted successful care integration and improved patient outcomes [24]. For example, the Croydon’s VW program in England has demonstrated the feasibility and effectiveness of delivering healthcare services in patients’ homes [25]. A focus group study exploring healthcare professionals’ experiences with interprofessional collaboration in VWs in Norway found that the VW model enables patient-centred care and a shared focus on health promotion for elderly patients with multimorbidity living at home [26]. Additionally, the use of remote patient monitoring technologies has gained prominence, particularly during the COVID-19 pandemic, as it strengthens healthcare delivery by enabling continuous monitoring and early detection of deterioration [20].

### 1.3. Swansea Bay Virtual Wards

In Swansea Bay, VWs have been created by the health board and are actively running in four out of the eight neighbourhood clusters. The system consists of acquiring suitable patients by identifying individuals through effective partnerships with primary care, secondary care, community care, and social care. Additionally, patients can be acquired through VW staff. After patients have been referred to the VW, they are assessed by a member of staff at their home, and they will be discussed at the weekly multidisciplinary team meeting to provide bespoke personal care. For each patient, a care plan will be created and agreed upon with a patient-individualised length of stay. The VW aims to provide holistic, high-quality, and patient-centred care through rapid assessment. Care can consist of physiological measures, phlebotomy, ECGs, occupational therapy, pharmacy reviews, and remote monitoring. Patient criteria include frailty, frequent attendance, admission avoidance, ‘long-stayer’ patients, palliative care, complex issues, falls, and chronic conditions. Patients are excluded from selection if they are acutely ill patients, undergoing bridging of care, and requiring routine GP follow-up.

In conclusion, VWs offer a promising approach to healthcare delivery, particularly for frail and elderly patients. The literature has emphasised the importance of predictive modelling, supply–demand matching, and efficient routing and scheduling in optimising resource allocation and improving patient outcomes in VWs. These advancements in telemedicine provide opportunities to meet the growing demand for healthcare services and enhance the quality of care provided to patients by treating them in their own homes. Further research and implementation initiatives are needed to fully realise the potential of VWs in improving healthcare delivery.

## 2. Methodology

### 2.1. A Virtual Ward Location Planning Model

Our mathematical model considers a VW system that provides remote monitoring services to elderly and frail patients at home across the entirety of Wales. The objective is to determine the optimal locations for VW hubs, the allocation of patients per output code to these VWs, and the number of clinicians with different skill levels required at each location, while still considering the cost of individual remote monitor kits for patients and the cost of said clinicians. The model is a mathematical optimisation problem that aims to determine the optimal locations for VWs and to allocate patients to these VWs; we also aim to determine the number of clinicians required at each level and each VW location. The considerations include the cost of the remote monitoring kit, the cost of the clinicians, and the cost of the startup. This model is created from the ideology of the one-step warehouse location problem, which is a well-known problem within operational research. The problem involves determining the best possible position for a facility to be built while also minimising the cost of supplying goods or a service to the set of demand points. One-step refers to the part of the problem that assumes every facility opens at the same time and that there is no uncertain demand or any other uncertainty. The problem is formulated as a mixed-integer linear programming problem. The decision variables are binary and continuous, representing the assignment of patients to VWs, the employment of clinicians at each VW, and the number of remote monitor kits for each VW.

### 2.2. Sets and Indices

Let I represent the set of potential VW hubs and J represent the set of patient demand points aggregated by postcode areas. Let K represent the set of different levels of clinicians available for remote monitoring services.

### 2.3. Parameters

The following parameters are defined. Let $f_{i}$ denote the fixed setup cost of opening a VW hub at location i∈I. Furthermore, let $d_{j}$ denote the demand of patients per output code j∈J for VWs and remote monitoring services. Let $Q_{i}^{staff}$ and $Q_{i}^{kits}$ be the staffing and kit capacity, respectively, of VWs at location i∈I in terms of the number of patients they can serve. Let $c_{k}$ be the skill level of each clinician k∈K. Let $h_{k}$ denote the cost of employing a clinician of level k∈K at a VW location. Finally, we introduce *r*, which is the cost of an individual remote monitoring kit for patients.

### 2.4. Decision Variables

The following decision variables are defined. We define $x_{ij}$ as a binary variable that equals 1 if a VW at location *i* serving patients with demands coming from output code *j* is deployed; otherwise, its value is 0. We furthermore introduce $y_{ij}$, which is a non-negative continuous variable indicating the number of remote monitoring kits allocated to patient demand point *j*, which is being operated at VW hub location *i*. Finally, we introduce $z_{ik}$ as a non-negative continuous variable indicating the number of clinicians of level *k* that are to be employed at VW location *i*.

### 2.5. Objective Function

The objective function (1) minimises the total cost.

(1) $$\mathrm{Minimise}\sum_{i\in I}\sum_{j\in J}f_{i}x_{ij}+\sum_{i\in I}\sum_{j\in J}ry_{ij}+\sum_{i\in I}\sum_{k\in K}h_{k}z_{ik}$$

This includes the setup costs for the virtual wards plus the individual setup cost for the patients’ remote monitoring kits and the costs of employing clinicians at virtual ward locations.

### 2.6. Constraints

The following constraints are introduced:

(2) $$\begin{array}{ccc} \sum_{i\in I}x_{ij}=1 & & \forall j\in J \end{array}$$

(3) $$\begin{array}{ccc} \sum_{j\in J}d_{j}x_{ij}\leq \sum_{k\in K}z_{ik}Q_{i}^{staff} & & \forall i\in I \end{array}$$

(4) $$\begin{array}{ccc} \sum_{j\in J}d_{j}x_{ij}\leq Q_{i}^{kits} & & \forall i\in I \end{array}$$

(5) $$\begin{array}{cccc} \; & x_{ij}\in \left\{0,1\right\} & & \forall i\in I,j\in J \end{array}$$

(6) $$\begin{array}{ccc} y_{ij}\geq 0 & & \forall i\in I,j\in J \end{array}$$

(7) $$\begin{array}{ccc} z_{ik}\geq 0 & & \forall i\in I,k\in K \end{array}$$

Constraints (2) ensure that all the output codes, i.e. regions, are covered, while (3) and (4) ensure that the virtual wards are staffed without exceeding capacity. (5)–(7) are the decision variables and their domains.

### 2.7. Model Assumptions and Parameter Setting

Within the model, it will be assumed that all patients are homogeneous, i.e. they do not exhibit scenario-based behaviours. Furthermore, we assume that the cost of the clinician in each skill bracket is homogeneous. Finally, the number of patients does not have to be an integer due to the model working on a yearly basis.

We assume that individual remote monitoring kits costs GBP 550 each. This kit includes an oximeter, thermometer, blood pressure cuff, table, and case, as well as the system it uses to operate. Demand is based on population data, and patients needing a virtual ward have a 14-day length of stay. In terms of clinician capacity, we assume that clinicians are able to visit 10 patients per day.

In terms of the setup cost, we base our calculations on numbers gained from the team for Swansea Bay VW and the different levels of clinicians. We can calculate roughly how much it would cost for each VW to be set up. Within the teams at Swansea Bay VWs, work includes two general practitioner (GP) sessions a month and two geriatrician sessions a month. These are both assumed to cost GBP 1000 per month. Additionally a Band 7 nurse and a Band 6 occupational therapist (OT) are required. These cost GBP 43,742 and 32,306 at entry level, respectively (NHS Employer, no date). The team also includes a Band 6 pharmacist, which incurs a starting salary cost of GBP 32,306. Then, a Band 4 assistant practitioner (AP) and Band 3 healthcare support worker (HCSW) are also required, which cost a salary of GBP 25,147 and 30,279 respectively. Lastly, the team also includes a Band 3 administrator, which costs a salary of GBP 22,816. As they are all employees of the NHS, we can assume these salaries are the same throughout Wales. Combining the cost of the whole team, the first year expenditure for the team would be GBP 210,596. Swansea Bay only operates in half of the clusters in the health board, so assuming that it needed to fund the whole health board, this number would have to be doubled, making it GBP 421,192. This cost does not include any equipment, remote monitoring kits, or transport facilities.

## 3. Results

### 3.1. Likely Demand Solution

The model was implemented on the demand level of the likely demand scenario. This took the 5% level of the elderly population in each output code. Using the address associated with the output codes in Wales, there were 183 locations. The algorithm has to compare 33,489 pairs of locations as all demand points are considered VW hubs. This takes a long time to run on the computer due to the high volume of pairs.

**Figure 1:**
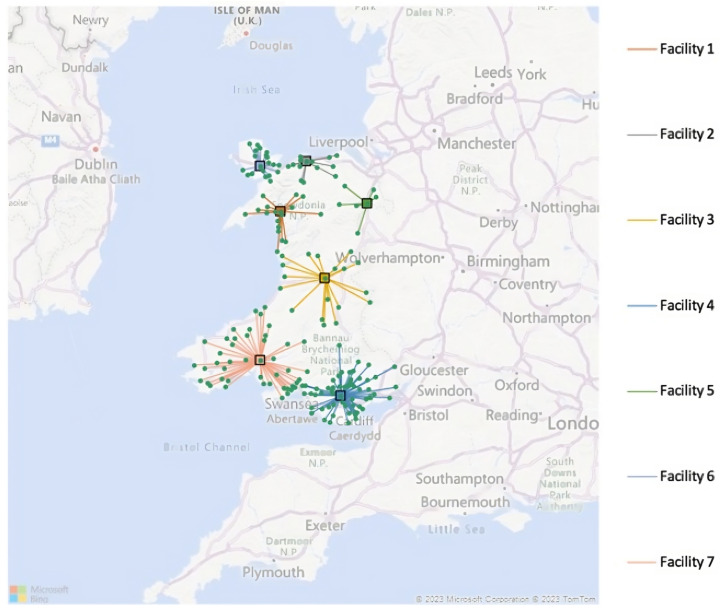
Likely demand solution of the virtual ward deployment problem.

The algorithm was programmed to find exactly seven facilities for the VW hubs to represent the seven health boards within Wales, with a service limit of 300 and a coverage distance limit of 200 miles [27]. Using the shortest car distance to minimise the cost, the algorithm ran using Bing Maps Key distances in miles. For this algorithm, the central processing unit time limit was 3720 s; the algorithm ran for 4739 iterations. The facilities were located at Penrhyndeudraeth, Mochdre, Llanidloes, Pontypridd, Ruabon, Llangefni, and Carmarthen, with their output codes listed as follows: LL48, LL28, SY18, CF37, LL14, LL77, and SA31. These represent the locations of the VW hubs that are the shortest distance away for clinicians accessing patients when needed. The outputs that are assigned to each hub location are listed below. For this deterministic solution, the total cost of establishing VWs across Wales would be GBP 3,211,234.14. The maximum distance a clinician would have to travel to a patient is 37.56 miles, and there would be a total of 15,925.2 patients in the system each year. The solution is illustrated below in Figure 1.

If TEC Cymru require every individual patient to have an individual remote monitoring kit, they would need to purchase 613 remote monitoring kits to supply the entirety of Wales on the basis that each patient is only on the ward for 14 days. The unit of demand allocated for each facility refers to the number of patients that the facility can have on the wards in the year. In the 2022 study by Bodil B. Jørgensen. et al, it was found that only 50% of geriatric patients are computer users; this would be the approximate proportion who would adopt remote monitoring. Thus, 7962.6 patients would require assistance from a clinician each year. With this assumption, 30.65 clinicians would need to be hired across Wales to support the 50% of patients who are non-computer users. Due to a lack of literature and resources, the number of clinicians of each band level that would need to be employed has been omitted.

For each facility, there is a specific level of patient demand, number of remote monitoring kits, and number of clinicians. These levels are illustrated in Table 1.

### 3.2. Demand Variation Solutions

In what follows, we provide an experimental analysis including demand variation experiments.

#### 3.2.1. High-Demand Scenario

**Figure 2:**
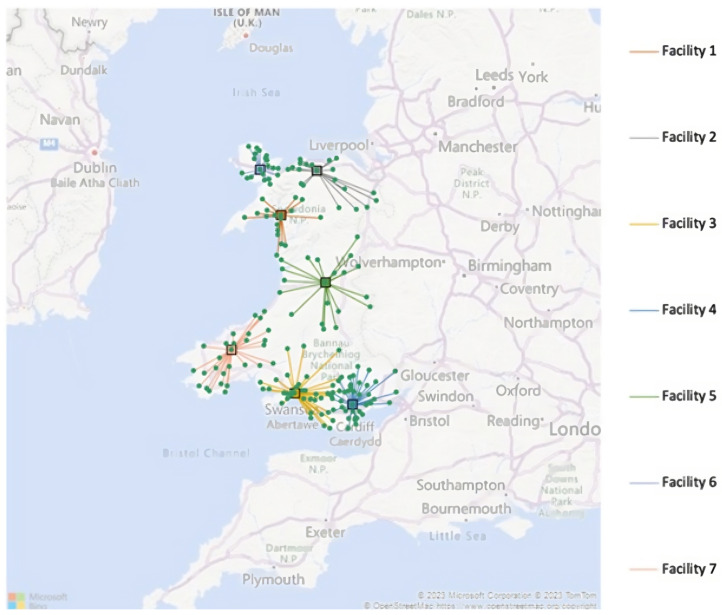
High-demand solution for the virtual ward deployment problem.

Using the demand points from Section 3.1 with 20% of the population, we ran the algorithm. It ran for 5248 iterations to locate the seven facilities. The solution is illustrated below in Figure 2.

The facilities were located at Penrhyndeudraeth, Betws-yn-Rhos, Llansamlet, Caerphilly, Llanidloes, Llangefni, and Crymych, with output codes of LL48, LL22, SA7, CF83, SY18, LL77, and SA36, respectively. These represent the locations of the VW hubs that are the shortest distance away for clinicians accessing patients when needed. The outputs that are assigned to each hub location are listed below.

The VW hubs could facilitate 31,850.40 patients a year. To cover the demands of these patients, 1225 remote monitoring kits would be purchased for patients staying for 14 days. With 50% being non-computer users, 15,925.2 patients would need to be assisted by a clinician each year. For those with high demands, 61.25 clinicians would need to be hired to meet the capacity of VWs. As in the likely demand solution, the band level of these clinicians would need to be later determined when there is more information on the requirement for a certain clinician.

#### 3.2.2. Low-Demand Solution

**Figure 3:**
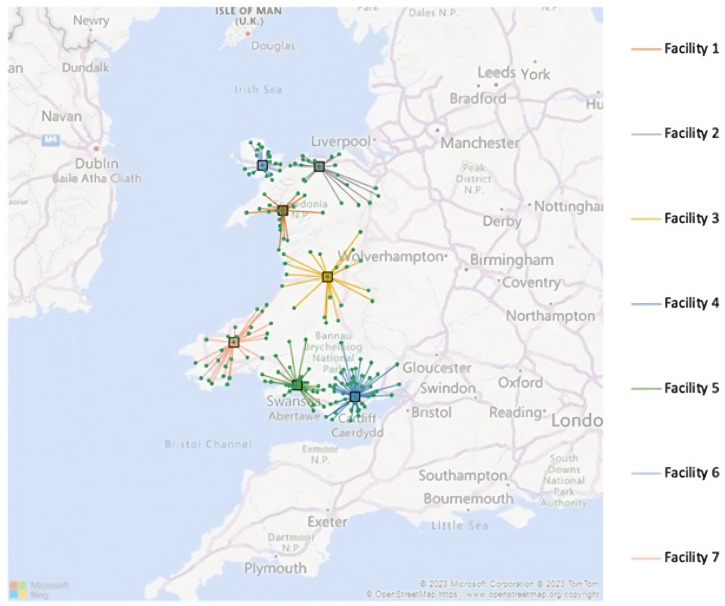
Low-demand solution of the virtual ward deployment problem.

Including all demand points in the algorithm, however, this time using demand of 1%, gave the following results. After running 6970 iterations, the tabu search located the seven facilities. The solution is illustrated below in Figure 3.

The facilities were located at Penrhyndeudraeth, Betws-yn-Rhos, Llanidloes, Caerphilly, Llansamlet, Llangefni, and Crymych, with the output codes listed as follows: LL48, LL22, SY18, CF83, SA7, LL77, and SA36. These represent the locations of the VW hubs that are the shortest distance away for clinicians accessing patients when needed.

The VW hubs could facilitate 3185.04 patients a year. To cover the demands of these patients, 123 remote monitoring kits would need to be purchased for patients staying for 14 days for the entirety of Wales. Additionally, 3185.04 patients would require assistance from a clinician each year. For this model, only 6.15 clinicians would need to be employed to cover the demand across Wales. As previously mentioned, the band level of these clinicians would need to be allocated depending on additional information regarding demand.

#### 3.2.3. Scenario Comparison

For both the high- and low-demand scenarios, the same seven facilities were located. However, the demand points assigned to these facilities are different. The comparison of the high- and low-demand scenarios, all facilities in central Wales and the north of Wales supply the same patient demand points in both scenarios. However, when demand is increased from low to high, the number of patient demand points are reduced in Caerphilly, with the two facilities in West Wales increasing in demand. Another observation is the increase in the service distance in comparison to the likely scenario. For both low and high demand, the maximum service distance was 37.56, which is slightly less than the likely demand with 37.74. It can also be seen that the facilities in both scenarios supply the same patient demand points and that the patient demand is just scaled up, as is to be expected.

Additionally, it is important to note that in all three scenarios, the output code LL48 consistently was assigned the same patient demand points. This finding suggests that regardless of the specific patient demand level, the VW hub would be allocated the same locations for its services. This location appears to be the most suitable and efficient choice for establishing a VW hub within that particular area.

For the likely demand solution, there are only three facilities with the same location as the high- and low-demand scenario. These are LL48, SY18, and LL77, representing Penrhyndeudraeth, Llanidloes, and Llangefni. The remaining four all changed their location. It is important to highlight that in this scenario, there are only two facilities in South Wales, while there are four facilities in North and Mid Wales.

After comparing the three scenarios, it would be advisable to open VW hubs in the following output areas: LL48, LL22, SA7, CF83, SY18, LL77, and SA36, all of which are locations for the high-demand and low-demand scenario. This is due to the three VW hubs located in South Wales, as it is more densely populated than North and Mid Wales. Thus, more patients would be treated per VW hub. The number of remote monitoring kits and clinicians would change with the demand and funding of the service.

### 3.3. Alternative Approaches

There are alternative ways of solving this model, including applying different optimising algorithms or heuristics. Due to the number of assumptions and the fact we are in the initial stages of research in this area, the approach used was most suitable. The usability of the FLP spreadsheet solver is at an accessible level for NHS staff working with VWs; therefore, any future changes that need to be made can be easily adapted. At a later stage in the projection of the project when there is additional information on setup cost for VWs, it can be easily edited.

## 4. Discussion and Limitations

Although we accomplished using quantitative modelling for planning virtual wards for frail and elderly patients, it is essential to acknowledge that the body of literature on planning for virtual wards (VWs) incorporating remote monitoring is small. As VWs represent a novel approach to traditional healthcare delivery, assumptions had to be made on the model’s parameters. Notably, assumptions regarding the homogeneity of patient behavior and the exclusive consideration of patients’ geographic distribution oversimplify the intricacies of patient demands and resource allocation within VWs. Consequently, there may be discrepancies between the assumed model and the reality of VW implementation.

Moreover, the study’s reliance on fixed-demand scenarios and expenses fails to account for dynamic factors such as inflation, technological advancements, and evolving patient needs, potentially compromising the model’s long-term predictive accuracy.

Additionally, our model demonstrates the use of different skill sets working in VWs. This is novel but applicable to real-world scenarios; the specific needs within VWs in each location would have to be dovetailed to the capacity built using the mathematical model.

Finally, the quality and availability of input data significantly influence the accuracy of the model’s outputs. However, using the scenario analysis can provide bounds on the number of staff, kit, and locations deployed, helping planners in the health service making analytics-driven decisions.

## 5. Conclusions and Future Work

In conclusion, our research will aid the Welsh Government with demand and capacity planning when introducing remote monitoring. The work examined the challenging and important topic of selecting the best VW sites and patient distribution while employing remote monitoring. In order to address the difficulties that healthcare systems confront in providing high-quality care to vulnerable populations, notably the frail and elderly, this study made use of operational research methodology integrated with the complex system in healthcare. The scenario analysis performed for this study further demonstrated the model’s flexibility and responsiveness to various demand scenarios, emphasising its practical usefulness in real-world circumstances. The model’s capacity to dynamically change resource allocation and produce the best patient care results was demonstrated by the distinction between likely, high- and low-demand situations.

This paper provided a first mathematical model to optimising the location, staffing, and equipment planning of VW hubs across Wales. The areas to explore in this project are as follows. In future work, including different cases of patients could be a way of expanding the model. This paper focused on frail and elderly patients; however, additional age categories (e.g., children) or clinical conditions (e.g., cancer) could be incorporated into the model. This may potentially change the output of the model, including VW locations and staffing.

## Figures and Tables

**Table 1:** Patient demand, number of remote monitoring kits and number of clinicians assigned to each location.

Post Code Area	LL48	LL28	SY18	CF37	LL14	LL77	SA31
Patient demand	1665.50	1285.90	1599.85	5225.50	606.55	1518.85	4023.05
Remote monitoring kits	64	50	62	201	23	58	155
Clinicians	3.2	2.5	3.1	10.05	1.15	2.9	7.75

## Data Availability

Data is available upon request.
